# A customized home-based computerized cognitive rehabilitation platform for patients with chronic-stage stroke: study protocol for a randomized controlled trial

**DOI:** 10.1186/s13063-018-2577-8

**Published:** 2018-03-22

**Authors:** Macarena Gil-Pagés, Javier Solana, Rocío Sánchez-Carrión, Jose M. Tormos, Antonia Enseñat-Cantallops, Alberto García-Molina

**Affiliations:** 1 Institut Guttmann, Institut Universitari de Neurorehabilitació, adscrit a la Universitat Autònoma de Barcelona, Badalona, Barcelona, Spain; 2 Universitat Autònoma de Barcelona, Bellaterra, Cerdanyola del Vallès, Spain; 3 Fundació Institut d’Investigació en Ciències de la Salut Germans Trias i Pujol, Badalona, Barcelona, Spain; 4 Departament de Psicologia Clínica i de la Salut, Universitat Autònoma de Barcelona, Bellaterra, Cerdanyola del Vallès, Spain

**Keywords:** Stroke, Chronic, Randomized controlled trial, Cognitive impairment, Computerized cognitive rehabilitation

## Abstract

**Background:**

Stroke patients usually suffer primary cognitive impairment related to attention, memory, and executive functions. This impairment causes a negative impact on the quality of life of patients and their families, and may be long term. Cognitive rehabilitation has been shown to be an effective way to treat cognitive impairment and should be continued after hospital discharge. Computerized cognitive rehabilitation can be performed at home using exercise programs that advance with predetermined course content, interval, and pace. We hypothesize that computerized rehabilitation might be improved if a program could customize course content and pace in response to patient-specific progress. The present pilot study is a randomized controlled double-blind crossover clinical trial aiming to study if chronic stroke patients with cognitive impairment could benefit from cognitive training through a customized tele-rehabilitation platform (“Guttmann, NeuroPersonalTrainer”^®^, GNPT^®^).

**Methods/design:**

Individuals with chronic-stage stroke will be recruited. Participants will be randomized to receive experimental intervention (customized tele-rehabilitation platform, GNPT^®^) or sham intervention (ictus.online), both with the same frequency and duration (five sessions per week over 6 weeks). After a washout period of 3 months, crossover will occur and participants from the GNPT^®^ condition will receive sham intervention, while participants originally from the sham intervention will receive GNPT^®^. Patients will be assessed before and after receiving each treatment regimen with an exhaustive neuropsychological battery. Primary outcomes will include rating measures that assess attention difficulties, memory failures, and executive dysfunction for daily activities, as well as performance-based measures of attention, memory, and executive functions.

**Discussion:**

Customized cognitive training could lead to better cognitive function in patients with chronic-stage stroke and improve their quality of life.

**Trial registration:**

NCT03326349. Registered 31 October 2017.

**Electronic supplementary material:**

The online version of this article (10.1186/s13063-018-2577-8) contains supplementary material, which is available to authorized users.

## Background

Stroke, the most common cerebrovascular disease, is a focal neurological disorder of abrupt development due to a pathological process in blood vessels [1]. There are three main types of stroke, namely transient ischemic attack, characterized by a loss of blood flow in the brain and which reverts in less than 24 h without associated acute infarction [2]; ischemic stroke, characterized by a lack of blood reaching part of the brain due to the obstruction of blood vessels and causing tissue damage (infarction), wherein cells die in the immediate area and those surrounding the infarction area are at risk; and a hemorrhagic stroke, where either a brain aneurysm bursts or a weakened blood vessel leaks, resulting in blood spillage into or around the brain, creating swelling and pressure, and damaging cells and tissue in the brain [3].

In 2013, according to the World Health Organization (WHO) and the Global Burden of Disease study, worldwide, there were 11–15 million people affected by stroke and almost 1.5 million deaths from this cerebrovascular disease [4, 5]. Moreover, in 2013, the total Disability-Adjusted Life Years (years of healthy life lost while living with a poor health condition) from all strokes was 51,429,440. In Spain, in 2011, the National Institute of Statistics reported 116,017 cases of stroke, corresponding to an incidence of 252 episodes per 100,000 inhabitants [6]. Although stroke incidence increases with advancing age, adults aged 20–64 years comprise 31% of the total global incidence.

Stroke often results in cognitive dysfunction, and medical treatment may cause great expense on a personal, family, economic, and social level. Depending on the area of the brain affected and the severity of lesions, stroke patients may suffer cognitive impairment, and alteration in emotional and behavioral regulation [7]. Generally, cognitive impairment derived from stroke includes alterations in attention, memory, and executive function [8].

Recent reports have begun to show positive results from the use of computerized cognitive rehabilitation systems (CCRS) for stroke patients to improve attention, memory, and executive functions. Nevertheless, more research is needed to better control variables and improve training designs in order to reduce heterogeneity and increase control of the intensity and level of performance during treatments [9–12].

CCRS allow adjustment of the type of exercises administered to the specific cognitive impairment profile of each patient, but within a fixed set of possible exercises such that heterogeneity of therapy choice is minimized. This can improve studies by allowing better categorization of patient groups that execute similar training sessions in a similar range of responses [13]. Further, CCRS offers the possibility of applying cognitive rehabilitation at home, while patient adherence and performance can be monitored online, so that patients do not need to live near, lodge near, or travel to a rehabilitation center to receive therapy. Because CCRS therapy is entirely digitized, it generates objective data that can be analyzed to determine the relative effectiveness of these interventions. We hypothesize that by allowing a trained professional to oversee an automated customization program that stratifies the level of difficulty, duration, and stimulus speed of presentation, we will reduce the heterogeneity of traditional cognitive training and improve the efficacy of intervention in chronic stroke patients.

The first objective of this pilot study is to assess if chronic stroke patients with cognitive impairment could benefit from cognitive training through a customized tele-rehabilitation platform (“Guttmann, NeuroPersonalTrainer”^®^, GNPT^*®*^) [14] intended to increase the control of experimental variables (cognitive impairment profile, adherence, and performance) traditionally identified as a source of experimental heterogeneity. The study aims to assess if this benefit could translate into an improvement of the trained cognitive domains (attention, memory, and executive functions).

The second objective is focused on generalization, namely the ability to use what has been learned in rehabilitation contexts and apply it in different environments [15]. Transfer of learning is included within the concept of generalization when specifically referring to the ability to apply specific strategies to related tasks [16]. Two types of transfer have been proposed – near transfer and far transfer [17]. By near transfer we mean that, through the training of a task within a given cognitive domain, improved function in other similar, untrained tasks may be observed in the same cognitive domain. For instance, a patient who performs selective attention exercises and improves execution through the training might improve their performance in other selective attention exercises too. By far transfer we mean that training in a given cognitive domain may improve performance of tasks in other cognitive domains. Such improvement will be observable in tasks that are structurally dissimilar from the ones used in the training. For instance, if a patient performs selective attention exercises, they may also improve their performance in memory tasks.

It has been demonstrated that computerized cognitive training can lead to the phenomenon of transfer, as previously studied in stroke patients [18]. Thus, our research aims to note whether the application of patient-customized tele-rehabilitation can give rise to an improvement in other functions that are based on cognitive domains related to those that have been trained (near transfer) as well as in different ones (far transfer).

Finally, the third objective is to assess the variables of demography (age, sex, years of education) and etiology (ischemic stroke or hemorrhage) and their impact on rehabilitation outcome, given the need to understand the patient characteristics that may influence treatment effectiveness [19].

## Methods

### Design

**Fig. 1:**
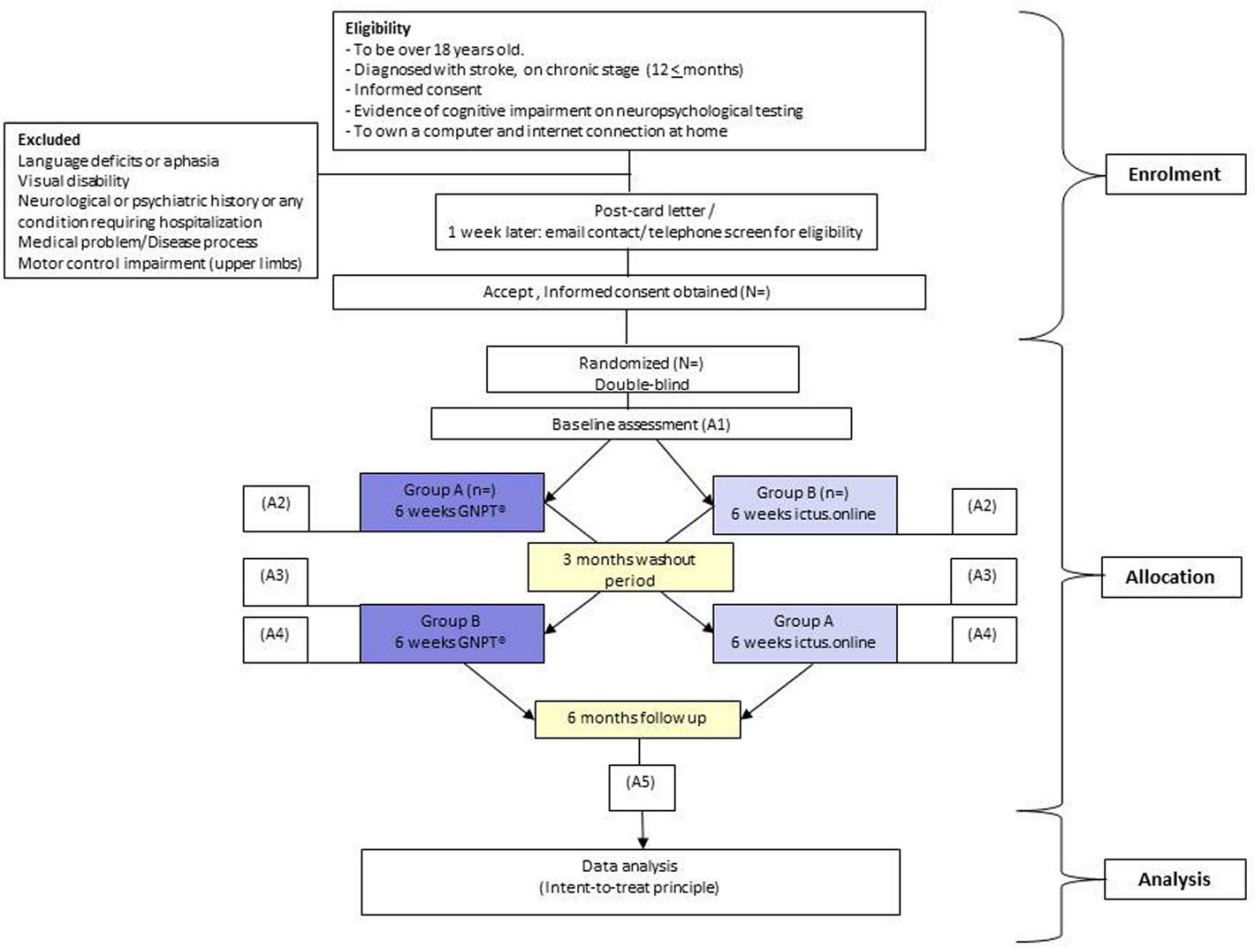
Study flow diagram

**Fig. 2:**
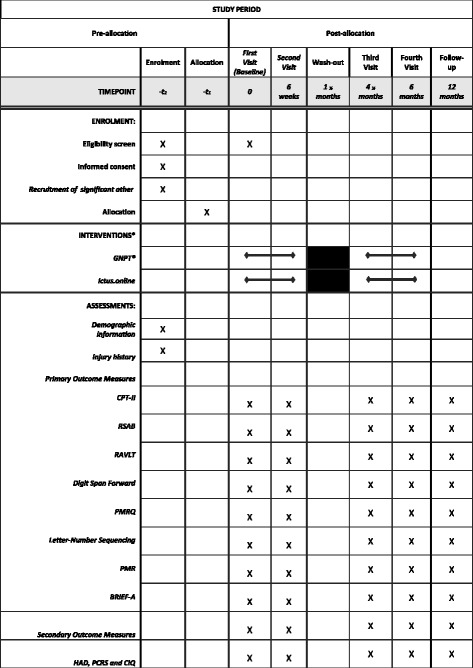
SPIRIT figure: schedule of enrolment, interventions and assessments

The present pilot study is a double-blind, randomized, crossover clinical trial with two arms (Figs. 1 and 2, SPIRIT). Participants will be randomly assigned to either experimental intervention (GNPT^®^) or sham intervention (ictus.online). In the first phase, participants in group A will start with the experimental intervention over a 6-week period (30 sessions), and participants in group B will start with the sham intervention over the same 6-week period (30 sessions). Participants will connect from their homes with their computer to the assigned intervention (experimental intervention or sham intervention). It will be indicated to them to connect once a day, from Monday to Friday, for 6 weeks. After a 3-month washout period, the second phase will commence, in which the groups will be crossed over, namely group A will receive the sham intervention and group B will receive the experimental intervention for 6 weeks each.

Evaluations will be conducted pre- and post-training by the study examiner. The examiner will be blind to the allocation of participants. Pre-testing will occur immediately before starting training (A1), and post-testing will occur immediately following both experimental and sham interventions (A2/A4) and after the 3-month washout period (A3). A follow-up neuropsychological assessment will be performed 6 months after treatment has terminated (A5).

A study researcher blinded to assessment procedure will perform the randomization of participants using the rand() function of Microsoft Excel software (Microsoft Excel 2010 for Windows), which is considered a good tool of randomization, having passed the Diehard test [20]. Each participant will have a “list entry” in our database, meaning that they will be stratified based on the two variables of interest in our study – sex (male or female) and type of stroke (ischemic or hemorrhagic). The rand() function will then assign random numbers from 0 to 1 to the stratified sample. The random numbers are ordered from lowest to highest and the first half of the list is assigned to group A and the second half to group B. A study researcher will create a letter with the information of the intervention to be performed (GNPT^®^ or ictus.online) for each participant and will place it in a sealed envelope. The examiner will deliver the sealed envelope to each participant when the first and the third neuropsychological examinations (A1 and A3) take place.

### Sample size

This is study will be an explorative pilot study. The total sample size required for a two-arm trial following some currently proposed approximation guidelines is between 24 and 70 [21]. Some recently published studies similar to this one have used sample sizes within this range [22–24]. The aim is to recruit 40 participants (*n* = 20 per group). The results of this pilot study will be used to compute sample size and conduct a power calculation to plan a full-scale study.

### Participants

Forty chronic-stage stroke patients with cognitive impairment involving alteration in attention, memory, and/or executive functions will be recruited. The sample will be composed of former patients of Institut Guttmann who have previously received cognitive rehabilitation in the sub-acute phase of evolution.

Recruitment will include postal, email, and telephone contact with participants. Participants will be informed about the study features, goals, and implications of their participation, the duration of the study and the type of procedures that will be applied. Informed consent will be obtained in order to include them in our database. After providing informed consent to participate, participants will schedule an appointment at our center for a baseline testing session.

Inclusion criteria for the trial are (1) age over 18 years old, (2) a stroke diagnosis (ischemic stroke or hemorrhagic stroke); (3) a time of evolution of 12 months or more since stroke occurred (chronic-stage); (4) cognitive impairment confirmed by pre-intervention neuropsychological assessment; and (5) willingness to give written informed consent to participate in the study. Exclusion criteria are (1) diagnosis of language deficits or aphasia; (2) motor impairment concerning upper limbs that may incapacitate them for computer use; (3) severe alteration of the visual field or perceptual problems; (4) health status that may require further intervention or admission to a medical center during the study; (5) neurological or psychiatric history; or (6) substance abuse.

A neuropsychological screen evaluation to check exclusion criteria (1) and (3) will be applied. It will consist of an orientation and language test (orientation, word-repetition, visual-naming, and order comprehension subtests of Test Barcelona-R) [25]. The cutoff needed to participate in the study is 100% proficiency on these tests.

In addition, we will recruit a next-of-kin for each participant, to whom we will administer questionnaires about the impact of attention, memory, and executive function impairment on the patient’s daily life at pre-training, post-training, and follow-up testing stages.

All recruited patients to date are able to consent and understand the implications of the study. Additionally, relatives are involved in both the process of patient consent and patient performance in the study. At the time of recruitment, next-of-kin have been included and informed of the study and its characteristics and implications. This reference next-of-kin collaborates in the study by completing questionnaires about how the participant functions in daily life.

### Ethical considerations and informed consent

Ethics approval has been received from the Care Ethics Committee of Fundació Institut Guttmann and the study will be conducted in accordance with the Declaration of Helsinki [26]. A neuropsychologist will explain the study to each participant by phone and in person. An information letter will be given to participants. Participants will be free to ask any question about study treatments and under no circumstances will study professionals imply that certain cognitive results will occur as a result of study participation. Further, a participant may resign from the study at any time.

The protocol follows the recommendations of the Standard Protocol Items: Recommendations for Interventional Trials Statement (SPIRIT) 2013 [27] (Additional files 1 and 2).

### Setting

The study requires that five neuropsychological assessments be held in a hospital setting at the Institut Guttmann. The Institut Guttmann is a hospital and academic institution located in Badalona (Barcelona, Spain). The hospital is specialized in the medical and surgical treatment and comprehensive rehabilitation of people with spinal cord injury, acquired brain injury, or other neurological disabilities. The institute’s main objective is to provide specialized, comprehensive, continuous, and personalized care, incorporating the highest levels of science and technology. In the case of patients with acquired brain injury, part of the rehabilitation includes cognitive rehabilitation, which is performed by neuropsychologists.

Participants will perform home-based interventions (GNPT^®^ and ictus.online).

### Intervention

#### Experimental intervention

GNPT^®^ is a tele-rehabilitation platform that allows therapists to configure and schedule rehabilitation sessions, consisting of a set of personalized computerized cognitive exercises. The examiner from the study will perform the initial neuropsychological assessment and the results of this assessment will be stored in the GNPT^®^ system by a study researcher. The program will then calculate a cognitive profile using these results, taking into account the patient’s age and educational level. Using this profile, the program will assign a set of computerized tasks to a certain day, configuring the input parameters of each task in order to personalize treatments. The neuropsychological assessment done at A1 (for group A) and A3 (for group B) will be used to establish the cognitive profile. Starting from each patient’s initial cognitive capacity, therapeutic plans will be adjusted to the level of execution and adapted to the patient’s evolution according to the obtained results. The adjustment is performed by GNPT^®^ using an automated process. The difficulty level of the tasks will vary and will be adapted according to the participant’s performance. Once a rehabilitation session is defined, the participant executes the assigned tasks, sending the results back to the server located at Institut Guttmann, such that therapists can asynchronously see the performance. A researcher from the study group will be exclusively dedicated to supervising this process.

**Fig. 3:**
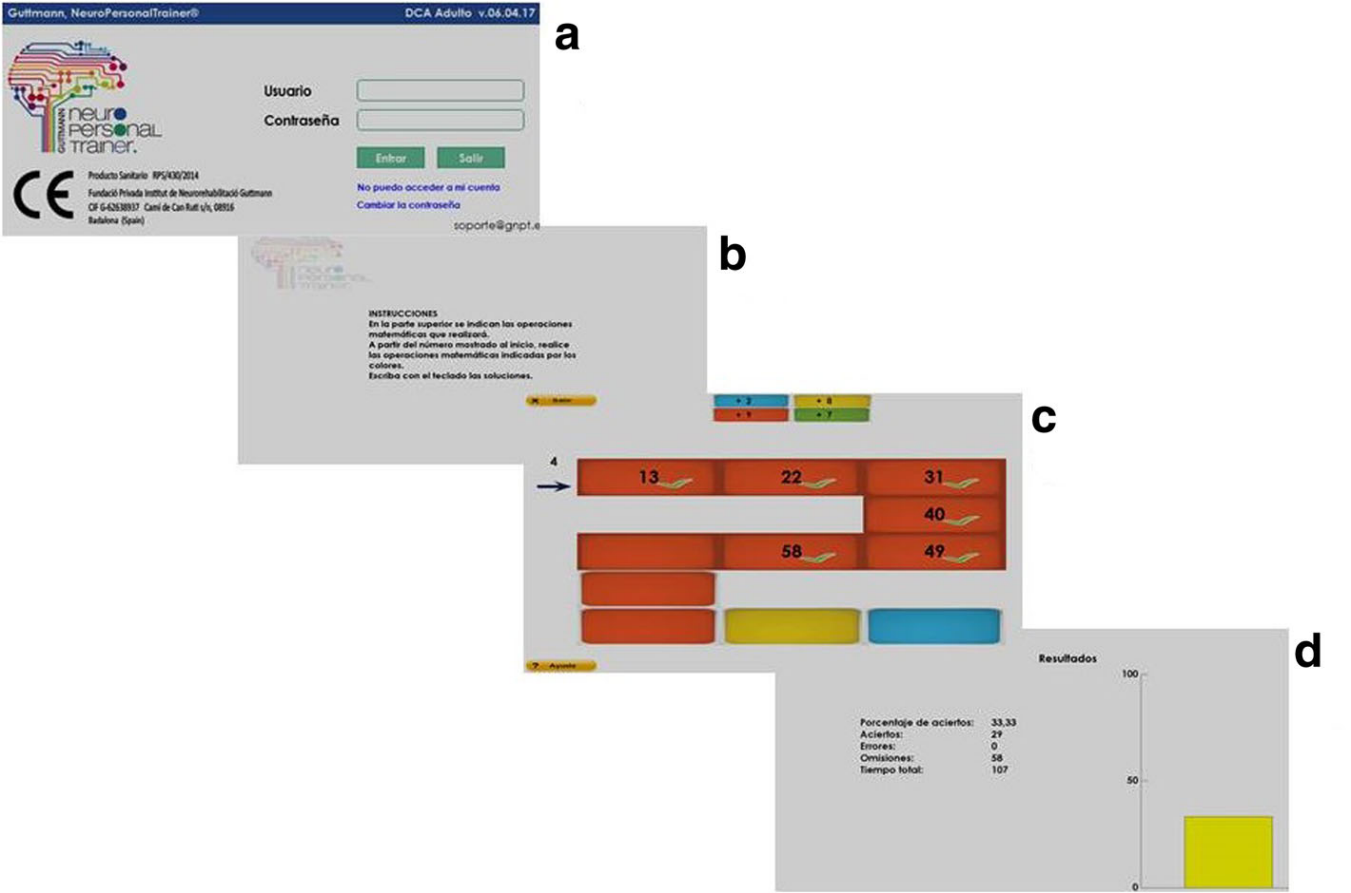
Experimental intervention (GNPT^®^) screenshots. **a** To access to the platform, the user must enter their username and password. **b** Each exercise begins with an instruction screen. **c** Example of a cognitive rehabilitation exercise. **d** At the end of each exercise, a results screen is displayed

The treatment consists of a set of 1-h sessions, five sessions per week during 6 weeks. A series of cognitive exercises of attention, memory, and executive functions will be conducted in each session (Fig. 3).

### Sham intervention

**Fig. 4:**
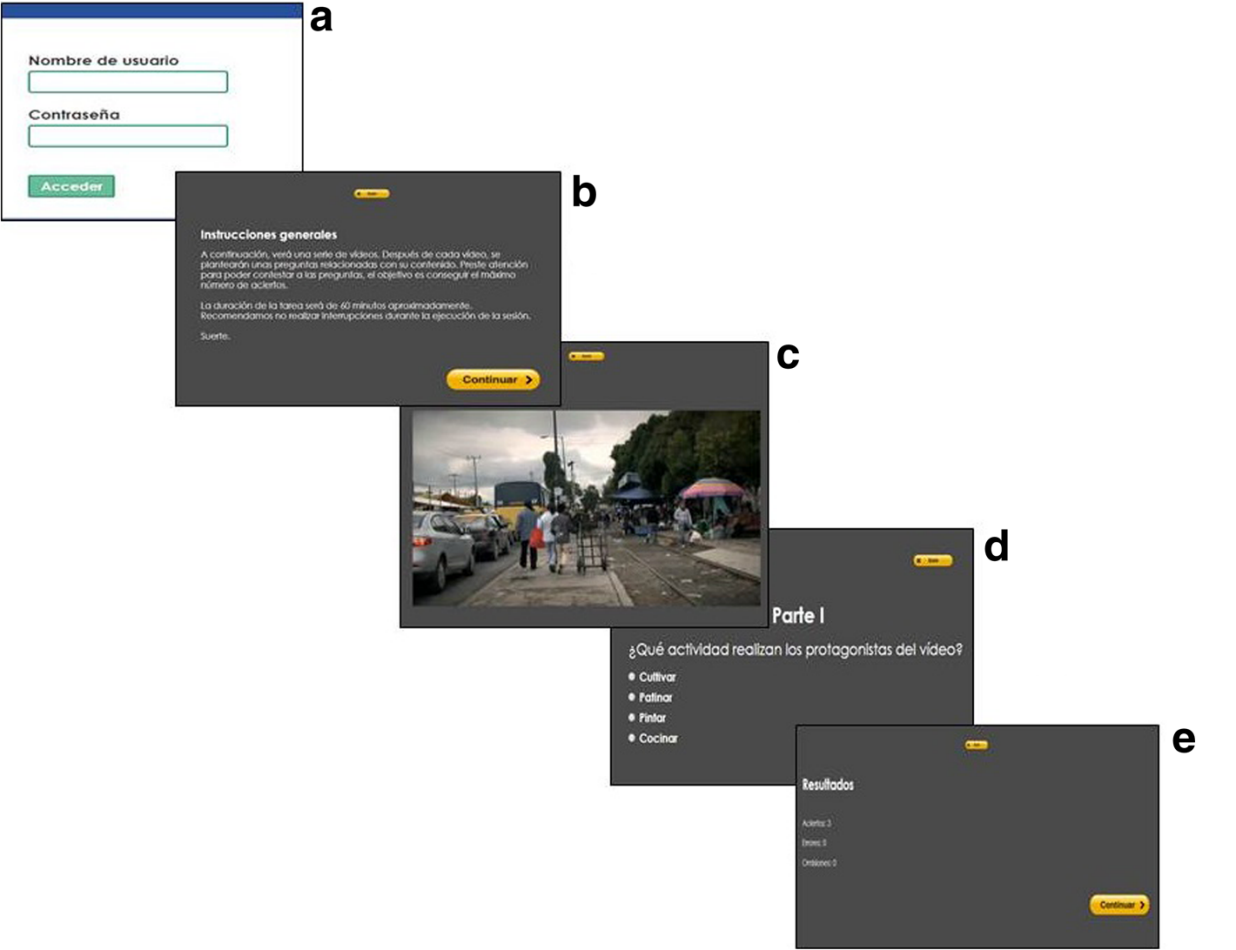
Sham intervention (ictus.online) screenshots. **a** To access to the platform, the user must enter their username and password. **b** Each exercise begins with an instruction screen. **c** The user watches a 10-min video. **d** When finished, the user accesses a three-question quiz with four response options. **e** When the quiz is finished, a results screen is displayed. In each session, three videos with their corresponding quiz are presented

For the sham intervention, a web platform has been developed and is available online through a the domain ictus.online (Fig. 4). This web platform has a graphic interface design similar to GNPT^®^. After login, it allows participants to access a daily session, which presents four videos of 10 minutes each. After each video, the participant must complete a three-question quiz about the contents of the video (for example, “What animal appears in the video?”). This makes each session last approximately 1 hour in total. The difficulty level of the questions is minimal and independent of the results of the neuropsychological assessment and will remain stable regardless of the execution of the participants. The quality of execution of this condition will be monitored.

Both the experimental intervention group and the sham intervention group will receive a username and password for logging in.

As adherence strategies, the organizers will offer flexible schedules to perform the exercises, phone calls to confirm appointment dates, and periodic progress reports to keep participants informed on their personal progress in the study (i.e., how much work they have completed, how much longer the study will last, etc.). Additionally, connection frequency will be supervised. To reduce risk, a member of the organizing team will contact the participants if they do not perform any exercise at any of the platforms of treatment for 3 consecutive days (GNPT^®^ or ictus.online) and will reinforce treatment compliance. Attrition will be considered when (1) presence of a new neurological condition appears during the study, or (2) loss of contact with participants occurs during the study and follow-up.

### Measures

Demographic information (age, sex, marital status, occupation, education level) and injury history (time of evolution, type of stroke) from all participants will be collected. Whether they have received or are currently receiving any kind of neuropsychological or functional rehabilitation at the moment of joining the study will be also recorded. The information collection method will be a personal interview. A comprehensive neuropsychological battery will be used to assess the participants. This battery is comprised of performance-based measures to assess attention (Conners Continuous Performance, CPT-II) [28], Trail Making Test A and B [29]), memory (Digit Span forward from the Wechsler Adult Intelligence Scale (WAIS-III) [30], Rey Auditory Verbal Learning Test [31]), speed of processing (Digit Symbol-Coding test from WAIS-III), visuoconstruction (Block Design test from WAIS-III), and executive functions (Digit Span backwards and Letter-Number Sequencing from WAIS-III, Stroop Color and Word test [32], Wisconsin Card Sorting Test – computerized version [33], and a Spanish phonemic fluency test – PMR [34]). Administered rating measures (Rating Scale for Attentional Behavior (RSAB) [35]; Prospective and Retrospective Memory Questionnaire (PRMQ) [36]; Behavior Rating Inventory of Executive Function – Adult Version (BRIEF-A)) [37] will be administered alongside the performance-based measures. All these rating measures will be applied to the patient (self-report) and their recruited next-of-kin (informant-report). Finally, a psychological well-being self-rating scale (Hospital Anxiety and Depression Scale [38]), Patient Competency Rating Scale [39], and the Community Integration Questionnaire [40] will be administered. The Hospital Anxiety and Depression scale will be used to detect participants with emotional disorders. The Patient Competency Rating Scale comprises 30 items, spanning a range of everyday situations and behaviors, and for each item, the patient must make judgment about their own level of competency. The Community Integration Questionnaire is a self-rating scale developed to measure community integration that comprises three subscales (home integration, social integration, and productive activities). These rating measures have been used before in other studies involving stroke patients [41–43].

#### Primary outcome measures

The primary outcome measures are performance-based measures and rating measures of attention, memory, and executive functions. Since they are different cognitive domains, we need to use different measures for attention (two primary outcomes: the CPT-II and RSAB), memory (Rey Auditory Verbal Learning Test, Digit Span forward from WAIS-III, and the PRMQ), and executive functions (Spanish phonemic fluency test – PMR, Digit Span backward, and the Letter-Number Sequencing Subtests from the WAIS-III and the BRIEF-A). CPT-II is a task-oriented computerized assessment of sustained attention; the Rey Auditory Verbal Learning Test assesses short- and long-term verbal memory and recognition; the PMR test assesses capacity of word generation according to an initial letter (P, M and R); the Digit Span forward test measures the span of immediate verbal recall; Digit Span backward and Letter Number assess working memory; and the RSAB comprises 14 items aimed to identify attentional difficulties on daily routines. The PRMQ includes 16 items, and was designed to evaluate prospective and retrospective memory in the short and long term. The BRIEF-A is designed to assess executive function behaviors on day-to-day activities.

In order to reduce the number of analyses, we will create composite cognitive indices – one for attention, another for memory, and another for executive functions, using sub-scores from the various performance-based measurements of the primary outcome. Composite cognitive indices will include sub-scores on CPT-II, namely number of omissions, number of commissions, and hit of response for attention; sub-scores on Rey Auditory Verbal Learning Test (Trials 1–5 recall, long-delay free recall and recognition) and Digit Span forward from WAIS-III (immediate recall of a series of numbers) for memory; and scores on Spanish phonemic fluency test – PMR (phonemic word fluency), Digit Span backward from the WAIS-III (immediate recall of a sequence of numbers in reverse order in which it was presented), and Letter-Number Sequencing Subtests from the WAIS-III (recall of progressively longer lists of intermixed letters and numbers in alphabetical and then numerical order) for executive functions.

#### Secondary outcome measures

Secondary outcomes are the Digit Symbol-Coding and the Block Design Subtests from the WAIS-III, Trail Making Test A and B (TMT-A and TMT-B), Wisconsin Card Sorting Test, and Stroop Color and Word Test. The Digit Symbol-Coding assesses the speed of processing; Block Design evaluates visuoconstruction and planning; TMT-A measures visual attention, while TMT-B measures task-switching; the Wisconsin Card Sorting Test is a measure of cognitive flexibility; and the Stroop Color and Word test is used as a measure of inhibitory control.

#### Statistical analysis

##### Analysis

Data analysis will be based on the intention-to-treat principle, which means that all participants who were randomized will be included in the final analysis, regardless of their adherence to any of the interventions. To avoid the problems derived from missing data (no attendance to neuropsychological assessments), blank spots will be filled with missing data imputation technique last value carried forward.

In order to detect changes in the variables recorded for the primary outcome and secondary outcome, a within-subject comparison pre-/post-intervention in both groups after both interventions will be made, as well as comparisons between groups (group A and group B).

Our null hypothesis is that a difference between baseline scores (A1 for group A and A3 for group B) and scores post-treatment (A2 for group A and A4 for group B) will not be significantly different from zero. Therefore, the Kolmogorov–Smirnov test will be used to examine the data for normality, and either ANOVA (parametric test) or Kruskal–Wallis (non-parametric test) will be used to analyze the measurements. A significance level of 95% will be used (*P* < 0.05 cutoff). Demographic (age, sex, years of education) and etiologic characteristics (ischemic stroke or hemorrhage) will be described using descriptive statistics for each group (group A and group B). The Bonferroni method will be applied to adjust the overall level of significance for multiple outcomes.

Alternatively, composite indices will be created by transforming raw neuropsychological test scores into z scores using published normative data. Domains will be created by averaging the z scores for each test within the domain (M = 0; SD = 1). This system has been used before in similar studies [44, 45].

If the sample size of this pilot study allows, a longitudinal mixed model will be applied.

Data analysis will be performed by an analyst outside the study with the statistical analysis software R and appropriate packages (“lmm” and “psych”) [46].

## Discussion

The first objective of this research is to study the effectiveness of a patient-customized computerized cognitive training program (GNPT^®^) as a home-based cognitive stimulation tool for stroke patients in the chronic stage of recovery. We hypothesize that training cognitive functions of attention, memory, and executive functions with GNPT^®^ could lead to better function of those cognitive domains post-therapy. Furthermore, we want to study whether customized cognitive training could lead to an improvement in other contexts different from the examination tests, improving performance in both related (near transfer) and unrelated (far transfer) tasks. Finally, we want to study the impact of variables such as demography and etiology on rehabilitation outcome.

The strengths of our study include that it is designed to (1) be easily replicated in new patient cohorts, (2) minimize unintended variability by reducing intervention heterogeneity, (3) adjust the type and difficulty of exercises to the specific cognitive impairment profile of patients, and (4) monitor adherence and performance of each exercise during the whole treatment.

Furthermore, our study focuses on targeting cognitive rehabilitation toward the improvement of the quality of life of the patient. In this sense, we include in our primary outcome measures an assessment of functioning in daily life, before, during, and after the performance of both interventions. Additionally, our design is longitudinal, which offers the possibility of studying the maintenance of the results over time (over 3 and 6 months). The timing of the primary outcome is 3 months. The purpose of the follow-up at 6 months is to check the evolution of patients once they have stopped receiving any type of intervention.

Furthermore, the execution of both interventions will be remotely monitored and we will be able to record the quantity and quality of the execution of the participants. Nevertheless, we consider a limitation the fact that we will not be able to control the environmental conditions in which participants perform the tasks. Another limitation is that the battery assessment will be the same throughout the study, which might create a learning effect and/or ceiling effect in some evaluation tasks.

## Trial status

This article was submitted on January 3, 2018. Recruitment is ongoing.

## Additional files

Additional file 1: SPIRIT 2013 Checklist: Recommended items to address in a clinical trial protocol and related documents*. (DOC 121 kb)

Additional file 2: CONSORT 2010 checklist of information to include when reporting a randomised trial*. (DOC 217 kb)

## Abbreviations

CPT-II: Conners Continuous Performance Test
A1: first assessment
A2: second assessment
A3: third assessment
A4: fourth assessment
A5: fifth assessment
BRIEF-A: Behavior Rating Inventory of Executive Function–Adult
GNPT^®^: “*Guttmann, NeuroPersonalTrainer”*^*®*^
PMR: Spanish phonemic fluency test
PRMQ: Prospective and Retrospective Memory Questionnaire
RSAB: Rating Scale for Attentional Behavior
TMT: Trail Making Test
WAIS: Wechsler Adult Intelligence Scale
